# No antidepressant-like acute effects of bright light on emotional information processing in healthy volunteers

**DOI:** 10.1007/s00213-021-06003-6

**Published:** 2021-11-06

**Authors:** Alexander Kaltenboeck, Tereza Ruzickova, Veronika Breunhölder, Tarek Zghoul, Philip J. Cowen, Catherine J. Harmer

**Affiliations:** 1 Department of Psychiatry, University of Oxford, Oxford, UK; 2 Clinical Division of Social Psychiatry, Department of Psychiatry and Psychotherapy, Medical University of Vienna, Vienna, Austria; 3 School of Mathematics and Maxwell Institute for Mathematical Sciences, University of Edinburgh, Edinburgh, UK; 4 Oxford Health NHS Foundation Trust, Warneford Hospital, Oxford, UK

**Keywords:** Bright light treatment, Phototherapy, Light therapy, Emotional information processing, Affective information processing, Antidepressant

## Abstract

**Rationale:**

Bright light treatment (BLT) is an efficacious antidepressant intervention, but its mechanism of action is not well understood. Antidepressant drugs acutely affect how emotional information is processed, pushing the brain to prioritise positive relative to negative input. Whether BLT could have a similar effect is not known to date.

**Objective:**

To test whether BLT acutely influences emotional information processing similar to antidepressant drugs, using an established healthy volunteer assay.

**Methods:**

Following a double-blind, parallel-group design, 49 healthy volunteers (18–65 years, 26 females) were randomly allocated to 60-min BLT (≥ 10,000 lux) or sham-placebo treatment early in the morning in autumn/winter. Immediately after treatment, emotional information processing was assessed using the Oxford Emotional Test Battery, a validated set of behavioural tasks tapping into emotional information processing in different cognitive domains. Participants also completed questionnaires before and after treatment to assess changes in subjective state.

**Results:**

The BLT group did not show significantly more positively biased emotional information processing compared to the placebo group (*p* > 0.05 for all measures). After adjustment for pre-treatment scores, there were also no significant post-treatment differences between groups in subjective state (*p* > 0.05 for all measures).

**Conclusions:**

BLT did not show immediate effects on emotional information processing in an established healthy volunteer assay. Thus, BLT might exert its clinical effects through a different (cognitive) mechanism than other antidepressant interventions. Future studies should corroborate this finding including clinical populations and more intensive treatment regimes, and control for potential chronobiological effects.

**Supplementary Information:**

The online version contains supplementary material available at 10.1007/s00213-021-06003-6.

## Introduction

Bright light treatment (BLT) is a non-pharmacological therapeutic modality whereby patients are exposed to high-intensity white light in order to mimic increased sunlight exposure. Although developed as a treatment for seasonal affective disorder (SAD), BLT has also shown efficacy in non-seasonal depression (Al-Karawi and Jubair 2016; Golden et al. 2005; Lieverse et al. 2011; Zhao et al. 2018; Zhou et al. 2018). Reported effect sizes seem comparable to those of commonly used antidepressant drugs (Golden et al. 2005), but BLT shows a faster onset of clinical effects and a more favourable side effect profile (Maruani and Geoffroy 2019; Oldham and Ciraulo 2014). Despite these advantages, the mechanisms through which BLT exerts its clinical effects are not well researched. A range of candidate mechanisms proposed so far include effects on the biological clock, melatonin secretion, central monoaminergic signalling (especially in mood regulatory areas, e.g. anterior cingulate or prefrontal cortex), and vegetative nervous system activity (Geoffroy et al. 2018; Maruani and Geoffroy 2019; Oldham and Ciraulo 2014; Pail et al. 2011).

In the context of antidepressant pharmacotherapy, a fruitful approach in the search for treatment mechanisms has included a focus on the processing of information with emotional valence (Harmer et al. 2017; Roiser et al. 2012). The “cognitive neuropsychological model of antidepressant treatment action” (CONEMATA) that resulted from this line of enquiry suggests that, regardless of their idiosyncratic neurobiological targets, antidepressants exert their clinical effects via a shared ability to push emotional information processing towards a preference for positive relative to negative input (thereby inducing a “positive bias”) (Harmer et al. 2017; Roiser et al. 2012; Warren et al. 2015).

For conventional antidepressant drugs, this model is well-supported by empirical evidence (for a comprehensive overview, see Warren et al. (2015)). The induction of positive biases following single-dose or short-term treatment has been consistently documented for various antidepressant drugs, in different cognitive domains, both in patients with depression and in healthy volunteers (Harmer et al. 2017; Roiser et al. 2012; Walsh et al. 2018a, b; Warren et al. 2015). Importantly, positive biases are generally observed in the absence of acute changes in subjective state (such as mood or anxiety), can be measured considerably earlier than symptom improvements in depression, and seem predictive of later-occurring clinical effects (Browning et al. 2019; Harmer et al. 2017; Warren et al. 2015).

Interestingly, the ability to induce positive biases in emotional information processing has not only been shown for antidepressant drugs, but also for several non-drug interventions, e.g. electroconvulsive therapy (Bai et al. 2017), transcranial direct current stimulation (Brunoni et al. 2014), high density negative ion treatment (Harmer et al. 2012), or cognitive behavioural therapy (Reinecke et al. 2013).

Whilst BLT seems to alter amygdala and prefrontal neural activity in response to negative emotional cues (Fisher et al. 2014), no study to date has assessed how BLT affects the processing of positive versus negative stimuli. Therefore, it is currently unknown whether BLT has valence-specific effects as predicted by the CONEMATA.

Drawing on an established experimental medicine assay, we assessed the effects of single-dose BLT on the processing of positive and negative stimuli in a range of cognitive domains. Immediate valence-specific effects on emotional information processing have been demonstrated for different antidepressant drugs as well as for some non-drug treatments (e.g. negative ion treatment, transcranial direct current stimulation) (Warren et al. 2015). In addition, there is observational evidence that serotonin turnover in the healthy human brain is closely linked to current sunlight exposure (Lambert et al. 2002). Furthermore, it has been demonstrated that blue light exposure in healthy volunteers can acutely alter brain activity in response to negative emotional vocal stimuli (Vandewalle et al. 2010). Taken together, these findings suggest that BLT could also show acute effects on behavioural measures of emotional information processing as would be predicted by the CONEMATA. We hypothesised that acute effects of BLT would parallel those of common antidepressant drugs, i.e. an induced shift towards a positive bias across cognitive domains, in the absence of changes in self-reported subjective state.

## Material and methods

### Study design and sample

The study used a between-group, randomised, double-blind, placebo-controlled design. It was approved by the university ethics board, and all participants gave written informed consent prior to inclusion. Fifty healthy volunteers were recruited, with sample size based on previous comparable studies, aiming for a power greater than 80% to detect a meaningful effect (Huneke et al. 2017; Walsh et al. 2018a). One participant was excluded from the final analysis because of prior experience with the study tasks. Thus, the final sample consisted of 49 participants, 26 females, aged 18 to 65 years (see Table 1 for details on demographic and psychological characteristics).

**Table 1:** Summary of demographic and psychological characteristics of treatment groups. Values represent group means with standard deviations in parentheses.

	Bright light (*n* = 25, 14 females)	Sham-placebo (*n* = 24, 12 females)
Age in years	24.7 (8.1)	28.6 (12.0)
Years in full-time education	17.2 (2.1)	17.8 (1.9)
IQ estimate English first language (Spot-the-Word Test)	111.5 (8.4)	115.9 (7.0)
IQ estimate English not first language (Spot-the-Word Test)	103.2 (7.4)	107.7 (6.4)
Self-reported estimate of hours outside on a typical day	2.6 (1.1)	3.0 (1.7)
Neuroticism (Eysenck Personality Questionnaire)	6.0 (4.8)	6.0 (4.5)
Psychoticism (Eysenck Personality Questionnaire)	2.4 (1.7)	2.5 (2.0)
Extraversion (Eysenck Personality Questionnaire)	15.2 (4.3)	13.2 (4.6)
Lie (Eysenck Personality Questionnaire)	9.6 (4.2)	9.4 (4.5)
Depressive symptoms (Beck Depression Inventory)	4.2 (4.5)	3.7 (3.8)
Trait anxiety (State-Trait Anxiety Inventory)	34.1 (8.6)	36.4 (10.0)

All participants underwent a structured psychiatric interview (SCID-5), a general medical interview, and assessment with the Eysenck Personality Questionnaire, Spot-the-Word Test, State-Trait Anxiety Inventory, and the Beck Depression Inventory II. Only volunteers who did not fulfil any of the pre-specified exclusion criteria (see supplements) were included.

### Experimental session

Testing took place between November and March, when sunlight exposure is low in the UK, with experimental sessions scheduled within 1 h after usual wake-up time. For female participants, following common practice, testing during pre-menstrual week was avoided. Before treatment, participants completed questionnaires assessing subjective state at baseline. Subsequently, they were randomly allocated to 60-min BLT or sham-placebo treatment in a double-blind protocol. Immediately following treatment, participants repeated the subjective state assessment and were asked about their expectations regarding the effects of the treatment received. Thereafter, they completed the Oxford Emotional Test Battery to assess emotional information processing.

### Treatments

BLT was applied using a Litepod Company Diamond 5 SAD light emitting full-spectrum white light (≥ 10,000 lux at 55 cm). Sham-placebo treatment was based on an established deception protocol whereby participants are told the aim of the trial was to compare two effective antidepressant interventions, BLT and negative ion treatment, but that half of all participants were going to receive a biologically inert placebo treatment that would appear like a genuine light or negative ion treatment but would not be expected to have a measurable effect (Lam et al. 2016). In reality, BLT always served as the active treatment, and a deactivated Bionaire air purifier (producing an audible hum and a mild air blow, but not emitting negative ions) always served as the placebo.

In order to ensure that both participants and researchers remained blind to the allocated treatment, the following approach was used: At recruitment and immediately before the experiment, participants were told the deception story outlined in the preceding paragraph. They were then led to a treatment room by the researcher where both treatment devices were set up in separate booths labelled with letters “A” and “B” (both treatment devices were visible to the participants). Participants were given a closed envelope that assigned them to either booth “A” or “B”. They were instructed to open the envelope once the researcher had left the room, then position themselves in front of their allocated treatment device within a marked distance (50 cm), and switch on the device by pressing a start button. They were told to keep their eyes open (except for blinking) throughout the whole session and not to sleep whilst receiving treatment. After 60 min, participants were notified through the door by the researcher that their treatment time was over. They were asked to switch off their device and put back the allocation note in the envelope so that the researcher would not know to which treatment booth they had been assigned. Participants were then asked to exit the room without telling the researcher which treatment they had received. This approach allowed us to run the whole experiment in a resource-efficient manner with neither the participant nor the researcher knowing whether an active or a sham-placebo treatment had been applied.

In order to ensure compliance with the instructions and adequate treatment in the absence of a researcher in the room, participants were video-recorded throughout the treatment session (they were informed about this at the beginning of the study). After all assessments were completed, the obtained video recording was checked by a researcher as a quality control measure. All participants who took part in the study complied with the instructions and received treatment of satisfactory quality.

### Subjective state assessment

Subjective state was assessed using the following questionnaires: Befindlichkeitsskala, State-Trait Anxiety Inventory, Positive and Negative Affect Schedule, and visual analogue scales of subjectively experienced emotions.

### Oxford Emotional Test Battery (ETB)

The ETB is a validated and widely used set of five behavioural tasks that allow assessment of emotional information processing in different cognitive domains. The tasks it comprises have been shown to be sensitive and specific to early effects of antidepressant drugs (see Warren et al. (2015) for a comprehensive overview). Participants complete the tasks in the order below.

#### Facial expression recognition task (FERT)

Participants are presented with pictures of human facial expressions of emotions. Each face displays one of six basic emotions (anger, disgust, fear, happiness, sadness, or surprise). Each emotional expression is presented at different levels of intensity (10%, 20%, 30%, 40%, 50%, 60%, 70%, 80%, 90%, and 100%), which have been created by combining shape and texture features of the two extremes “neutral” (i.e. 0%) and “full prototypical emotion” (i.e. 100%) to varying degrees (based on a previously described procedure by Young et al. (1997)). In total, 4 examples of each emotion at each intensity level are presented. Emotions are displayed by 10 different individuals overall, and for each of the 10 individuals, a neutral facial expression is presented as well. Thus, 250 stimulus presentations (6 emotions × 10 intensities × 4 examples + 10 neutral faces) are used in total. Facial expressions are presented in random order on a computer screen for approximately 500 ms each, followed by a blank black screen. Participants are instructed to correctly classify each facial expression as quickly and as accurately as possible. Responses are made by pushing one out of seven labelled buttons on a button box. Main outcomes of interest are hit rate, false alarm rate, non-identification rate, and median reaction time for correct classifications.

#### Emotional categorisation task (ECAT)

Participants are presented with positive and negative personality descriptors and are asked to correctly classify the valence of each word. In total, 60 words describing either extremely agreeable/positive characteristics (e.g. “cheerful”, “honest”, “optimistic”) or extremely disagreeable/negative characteristics (e.g. “domineering”, “untidy”, “hostile”) are presented individually in the centre of the screen for approximately 500 ms each. Positive and negative words have been chosen to be comparable with regard to frequency, length, and meaningfulness and are presented in the task in random order. Participants are instructed to imagine themselves overhearing someone describing them with each of the words and to indicate as quickly and accurately as possible whether they would like or dislike to be described with each of the words. Responses are made by pressing correspondingly labelled buttons on a button box. The main outcome of interest is the median reaction time for correct classifications.

#### Emotional faces dot probe task (FDOT)

Vigilance to emotional stimuli is assessed by comparing behavioural responses to a probe replacing a positive, negative, or neutral emotional cue. Each trial starts with the presentation of a fixation cross in the centre of the screen. This is followed by the presentation of a pair of pictures of facial expressions (neutral and neutral, neutral and fearful, or neutral and happy). One face appears above and the other one below the fixation cross. After approximately 100 ms, both faces disappear, and two dots in either vertical (:) or horizontal (..) orientation appear behind one of the faces. Participants are asked to indicate as quickly and as accurately as possible which orientation the dots are in. Half of all trials are masked, i.e. faces are presented on the screen for approximately 16 ms and are then replaced by a jumbled face for approximately 84 ms. The other half of trials are unmasked with faces simply being presented on the screen for approximately 100 ms. For neutral-emotional pairs, the emotional expression appears equally often on top and below the fixation cross, and the probe appears equally often behind the emotional and behind the neutral face. In total, the task consists of 192 trials (32 happy and neutral, 32 fearful and neutral, 32 neutral and neutral, once masked and once unmasked). The main outcome of interest is the vigilance score for emotional stimuli. Vigilance scores are calculated for emotional pairs by subtracting median reaction times in congruent trials (i.e. the probe appears behind the emotional expression) from those in incongruent trials (i.e. the probe appears behind the neutral expression). If a participant shows for example an attentional bias *towards* positive emotional information, one would expect that, when presented with a happy and neutral face pair, they will identify the probe in congruent trials (probe in position of the positive face) faster than in incongruent trials (probe in position of the neutral expression), thus resulting in a vigilance score for positive information greater than 0.

#### Emotional recall task (EREC)

Following a distraction period (approximately 15 min of engagement in the FDOT), subjects are asked to recall as many words as possible from the emotional categorisation task. They are given 4 min to write down as many words as they can. Main outcomes of interest are numbers of correctly and incorrectly recalled words.

#### Emotional recognition task (EMEM)

Following the EREC, participants are presented with positive and negative personality descriptors on the computer screen and are asked to indicate for each word whether they have encountered it in the ECAT completed previously. All 60 personality characteristics featured in the categorisation task as well as 60 novel distractor words (equal frequency of positive and negative words) are presented in random order in the centre of the screen, each for approximately 500 ms. Participants are asked to indicate as quickly and as accurately as possible for each word whether they have encountered it in the previous task. Main outcomes of interest are hit rate, false alarm rate, and median reaction time for correct classifications.

### Assessment of subjective expectations regarding treatment effects

To assess credibility of the employed sham-placebo protocol, participants were asked to rate three statements concerning their expectations about positive, negative, and general treatment effects on a 5-level Likert scale ranging from “Strongly Disagree” to “Strongly Agree” (see supplements for details).

### Statistical analysis

Subjective state measures were compared between groups using one-way ANCOVAs with post-treatment score as dependent variable, group as independent variable, and pre-treatment score as covariate. ETB performance was compared between groups using two-way mixed ANOVAs with emotion/valence as within-subjects factor. Where the assumption of sphericity was broken, the Greenhouse-Geisser correction was used. In order to corroborate findings, a robust mixed-design ANOVA based on the 20% trimmed mean was conducted, and where conclusions differed from the standard ANOVA, another robust mixed-design comparison based on an M-estimator and a bootstrap was used for clarification (see Field and Wilcox et al. (2017; Field et al. 2012) for details). For the FERT neutral face condition, groups were compared using standard independent *t*-tests and a robust equivalent based on the 20% trimmed mean (Field and Wilcox 2017). Expectations about treatment effects were compared using exact Wilcoxon-Mann-Whitney tests. All *p*-values are reported uncorrected for multiple comparisons, minimising the chance of a type II error.

## Results

None of the subjective state measures differed between groups after treatment when adjusted for pre-treatment scores (*p* > 0.05 for all measures, also see Table 2).

**Table 2:** Measures of subjective state before and after treatment for the BLT and sham-placebo group, respectively. Values represent means with standard deviations in parentheses.

	Bright light group		Sham-placebo group		ANCOVA result
	Baseline	After treatment	Baseline	After treatment	
BFS	17.5 (13.7)	12.1 (16.2)	24.5 (18.9)	18.5 (16.0)	*F*(1,46) = 0.2, *p* = 0.67
STAI state	31.2 (8.1)	29.6 (7.4)	34.8 (11.2)	31.9 (7.4)	*F*(1,46) = 0.1, *p* = 0.82
PANAS positive	32.6 (8.6)	33.1 (8.5)	27.6 (9.8)	27.9 (9.3)	*F*(1,46) = 0.6, *p* = 0.44
PANAS negative	12.5 (2.5)	12.3 (2.6)	12.9 (4.0)	12.0 (2.6)	*F*(1,46) = 1.0, *p* = 0.33
VAS anxious	18.2 (20.3)	17.4 (21.7)	16.5 (23.5)	9.4 (16.5)	*F*(1,44) = 2.0, *p* = 0.16
VAS alert	61.8 (22.6)	62.1 (26.5)	53.8 (24.5)	47.5 (29.7)	*F*(1,44) = 1.3, *p* = 0.26
VAS happy	70.0 (18.3)	72.6 (18.1)	58.8 (20.7)	65.5 (16.4)	*F*(1,44) < 0.001, *p* = 0.98
VAS sad	16.9 (20.7)	10.2 (11.6)	15.7 (22.0)	11.1 (13.9)	*F*(1,44) = 0.3, *p* = 0.60
VAS angry	7.2 (6.1)	5.4 (4.4)	9.0 (17.8)	4.8 (4.2)	*F*(1,44) = 0.4, *p* = 0.53
VAS disgusted	4.5 (5.9)	3.6 (3.9)	6.0 (11.3)	2.4 (2.0)	*F*(1,43) = 1.6, *p* = 0.21 (excluding one outlier)
VAS afraid	8.9 (10.5)	5.5 (7.5)	9.9 (18.7)	6.2 (13.3)	*F*(1,44) < 0.001, *p* = 0.98

Concerning emotional information processing, analysis of FERT hit rates (pre-specified primary outcome) suggested that there was no significant main effect of treatment (*F*(1,47) = 0.8, *p* = 0.37) and no significant treatment × emotion interaction (*F*(3.1,144.8) = 0.4, *p* = 0.73). Likewise, for FERT false alarm rates, there was no significant main effect of treatment (*F*(1,47) = 0.1, *p* = 0.81) and no significant treatment × emotion interaction (*F*(3.8,180.9) = 1.0, *p* = 0.42). For FERT non-identification rates, there was also no significant main effect of treatment (*F*(1,47) = 0.9, *p* = 0.35), but there was a significant treatment × emotion interaction (*F*(3.8,178.1) = 3.1, *p* = 0.02). However, this interaction was not significant in either of the robust analyses (*p* = 0.30 and *p* = 0.81, respectively), and further inspection suggested a false-positive result in the standard ANOVA due to violation of distributional assumptions. For FERT reaction times, there was no significant main effect of treatment (*F*(1,47) = 0.9, *p* = 0.36) and no significant treatment × emotion interaction (*F*(1.9,90.5) = 0.6, *p* = 0.52). Hit rates (*t*(47) = 0.9, *p* = 0.37) and reaction times (*t*(47) = 0.3, *p* = 0.76) for neutral faces also did not differ between groups. Except for the aforementioned discrepancy in the non-identification rate, standard and robust analyses led to the same conclusions.^1^

No significant main effect of treatment or treatment × valence interaction was found for ECAT reaction times, FDOT vigilance scores, or EREC numbers of words correctly or incorrectly recalled (*p* > 0.05 for all measures). Similarly, analysis of hit and false alarm rates as well as reaction times in EMEM led to no significant main effect of treatment or treatment × valence interaction (*p* > 0.05 for all measures). For all ECAT, FDOT, EREC, and EMEM measures, standard and robust analyses yielded the same conclusions. Details on all analysis results can be found in Table 3 and 4.

**Table 3:** Summary of results of mixed-effects ANOVAs comparing ETB outcomes of bright light versus sham-placebo treatment.

Task	Outcome measure	Main effect of treatment	Treatment × emotion or valence interaction	Robust mixed ANOVA confirming result
FERT	Hit rate	*F*(1,47) = 0.8, *p* = 0.37	*F*(3.1,144.8) = 0.4, *p* = 0.73	Yes
	False alarm rate	*F*(1,47) = 0.1, *p* = 0.81	*F*(3.8,180.9) = 1.0, *p* = 0.42	Yes
	Non-identification rate	*F*(1,47) = 0.9, *p* = 0.35	*F*(3.8,178.1) = 3.1, *p* = 0.02	No
	Reaction time	*F*(1,47) = 0.9, *p* = 0.36	*F*(1.9,90.5) = 0.6, *p* = 0.52	Yes
ECAT	Reaction time	*F*(1,47) = 0.4, *p* = 0.54	*F*(1,47) = 0.7, *p* = 0.39	Yes
FDOT	Vigilance bias masked	*F*(1,47) = 0.1, *p* = 0.80	*F*(1,47) = 0.03, *p* = 0.86	Yes
	Vigilance bias unmasked	*F*(1,47) = 2.6, *p* = 0.12	*F*(1,47) = 2.0, *p* = 0.16	Yes
EREC	Hits	*F*(1,47) = 1.1, *p* = 0.30	*F*(1,47) = 0.04, *p* = 0.84	Yes
	Intrusions	*F*(1,47) = 0.001, *p* = 0.97	*F*(1,47) = 0.03, *p* = 0.85	Yes
EMEM	Hit rate	*F*(1,47) = 0.2, *p* = 0.65	*F*(1,47) = 0.1, *p* = 0.72	Yes
	False alarm rate	*F*(1,47) = 0.01 , *p* = 0.92	*F*(1,47) = 2.2, *p* = 0.15	Yes
	Reaction time	*F*(1,47) = 0.6, *p* = 0.46	*F*(1,47) = 0.2, *p* = 0.68	Yes

**Table 4:** Performance in the Oxford Emotional Test Battery for the BLT and sham-placebo group, respectively. Values represent means with standard deviations in parentheses.

	Bright light	Sham-placebo
Facial expression recognition task		
Hit rate [%]		
Anger	57.1 (11.3)	58.4 (13.2)
Disgust	64.7 (12.9)	60.1 (11.0)
Fear	52.5 (17.6)	50.5 (19.3)
Happiness	81.7 (6.7)	79.4 (5.6)
Sadness	66.3 (6.9)	64.4 (10.7)
Surprise	69.1 (5.8)	67.7 (5.9)
Neutral	80.0 (12.9)	83.3 (13.1)
False alarm rate [%]		
Anger	2.1 (2.0)	2.2 (1.9)
Disgust	2.0 (1.8)	3.1 (2.9)
Fear	1.3 (1.3)	1.3 (1.7)
Happiness	0.6 (0.7)	0.5 (0.7)
Sadness	3.1 (2.2)	2.6 (3.1)
Surprise	3.0 (2.6)	2.9 (2.6)
Non-identification rate [%]		
Anger	34.2 (7.7)	31.5 (9.0)
Disgust	21.7 (5.3)	25.3 (6.4)
Fear	22.0 (4.2)	22.6 (5.1)
Happiness	15.1 (5.8)	17.8 (3.3)
Sadness	31.5 (7.0)	32.1 (8.3)
Surprise	25.4 (4.9)	28.2 (6.1)
Reaction time [ms]		
Anger	1422.1 (309.4)	1495.3 (338.6)
Disgust	1480.6 (405.8)	1526.6 (276.3)
Fear	1877.3 (467.0)	2071.7 (835.3)
Happiness	1177.5 (234.9)	1181.7 (168.2)
Sadness	1220.6 (232.4)	1275.3 (154.8)
Surprise	1260.3 (243.9)	1289.5 (185.7)
Neutral	1204.8 (310.9)	1230.0 (250.3)
Emotional categorisation task		
Reaction time [ms]		
Positive	843.1 (169.6)	828.8 (133.3)
Negative	943.6 (191.4)	902.3 (170.5)
Faces dot probe task		
Vigilance bias score [ms]		
Masked positive	−9.7 (41.3)	−6.0 (32.9)
Masked negative	1.3 (48.3)	2.1 (46.6)
Unmasked positive	−4.3 (39.0)	19.6 (40.1)
Unmasked negative	6.1 (38.1)	8.2 (39.2)
Emotional recall task		
Hits [*n* correct]		
Positive	6.0 (3.1)	5.3 (3.1)
Negative	5.5 (3.3)	4.6 (2.6)
Intrusions [*n* wrong]		
Positive	2.3 (1.8)	2.3 (2.0)
Negative	1.5 (1.9)	1.5 (2.3)
Emotional recognition task		
Hit rate [%]		
Positive	83.6 (9.4)	85.8 (13.8)
Negative	72.1 (14.8)	73.1 (16.0)
False alarm rate [%]		
Positive	26.5 (14.9)	23.5 (16.5)
Negative	14.1 (8.9)	16.5 (11.7)
Reaction time [ms]		
Positive	904.7 (190.1)	938.0 (175.3)
Negative	1025.8 (195.7)	1072.5 (221.5)

Comparison of participants’ expectations about treatment effects yielded a significant group difference concerning a general effect (“*I believe that the treatment I have just undergone can in general influence a person’s emotions or mood (either positively or negatively)”*, *Z* = 2.4, *p* = 0.02). However, there were no significant differences when the direction of expected effects was explicitly specified as positive (*Z* = 1.5, *p* = 0.12) or negative (*Z* = 0.6, *p* = 0.57). Participants in the BLT group showed stronger expectations about a general effect on mood/emotion than participants in the sham-placebo group.^2^

## Discussion

We employed an established experimental medicine assay to test whether BLT can acutely induce a positive bias in emotional information processing similar to antidepressant drugs (and some non-drug treatments) (Harmer et al. 2017; Roiser et al. 2012; Warren et al. 2015). Contrary to our expectations, we did not observe such an effect.

There was no immediate valence-specific effect of BLT on emotional information processing in any cognitive domain. Whilst this result is preliminary and has to be replicated by future research, it might indicate that, at least on the cognitive level, BLT exerts its clinical effects via a different mechanism than many common antidepressant drug (and some non-drug) treatments and that the clinical effects of BLT might therefore not be explained within the theoretical framework of the CONEMATA. This, however, has to be interpreted considering that emotional information processing was assessed immediately after treatment and that only healthy volunteers were studied. Thus, it might be that BLT has indirect effects on emotional processing (e.g. by altering sleep or circadian rhythms) that only become apparent later on (LeGates et al. 2014). Similarly, BLT might show acute effects on emotional information processing only in certain patient populations (e.g. individuals suffering from SAD).

As predicted, BLT also showed no effect on subjective state. This observation is consistent with the characteristics of antidepressant drugs (Capitão et al. 2019; Walsh et al. 2018a; Warren et al. 2015). However, at least with regard to *acute effects*, it suggests scepticism towards common intuitions and claims about positive effects of bright (sun-)light on mood in healthy people. Further research concerning this question is warranted.

Paralleling previous observations (Desan et al. 2007; Eastman et al. 1998; Lam et al. 2016), we found that participants receiving sham negative ion treatment held comparable expectations about treatment effects as those receiving BLT—but only when the direction of the effect was explicitly specified. Interestingly, when asked whether they believed their assigned treatment had *any* impact on mood/emotion (i.e. no specified direction), bright light-treated participants exhibited stronger expectations for an effect. This could indicate that, at least in healthy volunteers, the employed placebo protocol using sham negative ion treatment and a cover story might not be as good a control as previously suggested in patients with depression (Lam et al. 2016). Alternatively, the protocol might have become less effective in inducing expectations comparable to BLT over time, e.g. due to increasing popularity and public awareness of the antidepressant effects of bright light. It has previously been demonstrated that the ETB is relatively robust against placebo effects (Huneke et al. 2017); therefore, this should not pose a significant problem in the context of our study (and the results did not change when expectation of a general effect was included as a covariate in the analysis). However, it is recommended that future studies employing similar sham-placebo protocols (especially in healthy volunteers) also explicitly assess treatment expectations to ascertain the validity of their protocol.

### Study strengths and limitations

The study was based on a clear pre-specified hypothesis derived from a large corpus of literature documenting valence-specific effects of antidepressant interventions on emotional information processing (Harmer et al. 2017; Roiser et al. 2012; Warren et al. 2015). The experimental medicine assay we employed is widely used and well-validated and has been shown to be *sensitive* and *specific* for acute and subacute antidepressant drug manipulations (Warren et al. 2015). In contrast to many other studies investigating mechanisms of light treatment, we explicitly refrained from preparing our participants with light deprivation prior to the experiment in order to keep the intervention as ecologically valid as possible (i.e. we studied potential effects of BLT such as patients would use the treatment). Finally, we employed an established sham-placebo protocol in order to create a credible control condition. However, because this protocol has been used largely in a clinical context, we also aimed to validate its usefulness in healthy volunteers in the context of an experimental medicine study by explicitly assessing participants’ treatment expectations.

Whilst our study is the first to provide empirical data concerning the question whether BLT might act via an acute influence on emotional information processing, the findings must be viewed in the context of several limitations.

First, BLT might have a weaker acute effect on emotional information processing than common antidepressant drugs. Though the study was adequately powered to detect large effects (as are typically observed in the ETB for single-dose and subacute treatments with antidepressant drugs (Huneke et al. 2017; Walsh et al. 2018a)), it was under-powered to reliably capture small effects. However, given that the clinical effects of BLT seem comparable in magnitude to those of antidepressant drugs (Golden et al. 2005; Lam et al. 2016), it is not clear why smaller effect sizes on emotional information processing would be expected and—perhaps even more important—whether they would be mechanistically relevant.

Second, we used only a single-dose treatment and assessed emotional information processing immediately thereafter. Given that light can also influence cognition through the indirect pathway of sleep and circadian phase changes (LeGates et al. 2014), it is possible that potential effects of BLT on emotional information processing only become apparent after a prolonged period of time or after repeated treatment, and hence would not have been captured in this study. Fisher et al. (2014), for example, treated healthy volunteers with bright light over a period of 3 weeks and found significant effects on amygdala and prefrontal neural activity in response to threat-related stimuli. It would thus be desirable to replicate this study assessing emotional information processing after a repeated BLT regime (e.g. 7 days of treatment) and to explicitly take into account potential chronobiological influences.

Whilst subjective state was assessed both at baseline and after the intervention, emotional information processing was tested only after treatment. This approach was based on a number of previous similar studies investigating acute effects of different drug (and non-drug) treatments on emotional information processing (Warren et al. 2015). The decision to assess emotion-related cognition only after treatment is based mainly on two considerations: First, running the test battery twice is relatively demanding for study participants, which can decrease engagement in the tasks and willingness to volunteer in the study. Second, repeated testing with the ETB can give rise to practice effects, whereby participants learn to respond most efficiently to cues prior to the intervention, which can subsequently decrease sensitivity to detect a meaningful treatment effect. Complicating matters further, the treatment itself could also influence the development of a practice effect.

Finally, we utilised a sample of healthy volunteers without any personal or family history of mental health problems. Although this population allows for a clearer assessment of treatment mechanisms unconfounded by psychopathological changes, it might be less sensitive to effects treatment. Future studies on effects of light on emotional information processing should therefore also include clinical populations (e.g. patients with seasonal affective disorder or non-seasonal depression). Furthermore, different (neuro)physiological and psychological effects of light exposure have previously been documented in healthy volunteers, including changes in autonomic activity (Sakakibara et al. 2000), serotonin turnover (Lambert et al. 2002), brain activity (Fisher et al. 2014; Macoveanu et al. 2016), and psychomotor vigilance (Phipps-Nelson et al. 2003). Although the focus of this study was on emotional information processing, measuring such additional biological effects could have aided the interpretation of the obtained null results (e.g. by highlighting how effects on different levels of neuroscientific description did (or did not) relate to effects on emotional cognition). Future trials investigating the effects of BLT on emotional information processing might therefore additionally obtain measures of neurotransmitter metabolism, brain activity, autonomic function, and cold cognition to more comprehensively understand the potential influence of light on the human mind and brain.

## Conclusion

BLT had no acute influence on subjective state or emotional information processing in an established experimental medicine assay of antidepressant treatment action. This finding might indicate that BLT works through a different (cognitive) mechanism of action than common antidepressant drugs. Future studies on this topic should include clinical populations, employ longer treatment regimes, and take into account potential chronobiological and sleep-mediated effects. Additionally, sham negative ion treatment in conjunction with a cover story needs further evaluation as a credible placebo control in healthy volunteers.

## Supplementary information

ESM 1 (DOCX 24.3 kb)

ESM 2 (PDF 164 kb)

## Footnotes

1 As an alternative approach, we also compared groups on signal detection theory measures (target sensitivity and response bias, calculated following Grier (1971)). This analysis also did not find a significant main effect of treatment or a treatment × emotion interaction.

2 Although the Oxford ETB has previously been shown to be robust against placebo effects (Huneke et al. 2017), all emotional processing analyses were repeated with expectations of a general effect included as a covariate in order to rule out any confounding effects. This led to the same overall conclusions as reported above.
