# Supplementary material for: No antidepressant-like acute effects of bright light on emotional information processing in healthy volunteers
Source: Psychopharmacology (Berl). 2021 Nov 6;239(1):277–86. doi: 10.1007/s00213-021-06003-6 (PMC8770384; doi:10.1007/s00213-021-06003-6)
Supplement: Supplementary file 1 — (DOCX 24.3 kb) [file 213_2021_6003_MOESM1_ESM.docx]

**Supplements to “No antidepressant-like acute effects of bright light on emotional information processing in healthy volunteers”**

# Exclusion criteria for the study

1. Any current or past major psychiatric disorder
2. Any first-degree relative with a diagnosis of schizophrenia-spectrum or other psychotic disorder, bipolar disorder, or depressive disorder
3. Any severe medical condition not stabilized at the time of the experiment (e.g., cardiac disease)
4. Any condition precluding treatment with bright light (e.g., retinal disorder)
5. Any current or past major physical illness that has the potential to significantly affect mental functioning (e.g., Parkinson’s disease)
6. Pregnant, lactating, or sexually active female who does not use a medically accepted method of contraception
7. Any history of seizures or any condition with the potential to manifest with seizures
8. Diagnosis of diabetes (risk of retinal disorder)
9. Current intake of medication that has a significant potential to affect mental functioning, or intake of such medication in the previous 3 months (e.g., antidepressants, neuroleptics, sedatives)
10. Any intake of recreational drugs in the last 3 months prior to the experiment
11. Excessive alcohol consumption up to 3 days before the experiment
12. Previous use of bright light treatment or negative ion treatment
13. Participant usually (more than 5 days a week) wakes up later than 10.00 a.m.
14. Necessity to wear tinted glasses
15. Any kind of sun exposure in the last month that would be expected to be unusually high compared to local conditions (e.g., beach vacation, skiing holidays)
16. Researcher’s decision to exclude a participant for any other reason (e.g., participation could be harmful to a participant, participant does not properly interact with the researchers at screening, participant has previous experience with Oxford Emotional Test Battery)

# Description of statements used to assess subjective expectations of treatment effects

Participants were presented with three separate statements about possible treatment effects:

(1) “*I believe that the treatment I have just undergone will influence my mood or emotions in a positive way or make me feel happier.*”

(2) “*I believe that the treatment I have just undergone will influence my mood or emotions in a negative way or make me feel less happy.*”

(3) “*I believe that the treatment I have just undergone can in general influence a person’s emotions or mood (either positively or negatively).*”
