# Supplementary material for: No antidepressant-like acute effects of bright light on emotional information processing in healthy volunteers
Source: Psychopharmacology (Berl). 2021 Nov 6;239(1):277–86. doi: 10.1007/s00213-021-06003-6 (PMC8770384; doi:10.1007/s00213-021-06003-6)
Supplement: Supplementary file 2 — (PDF 164 kb) [file 213_2021_6003_MOESM2_ESM.pdf]

Springer
the language of science

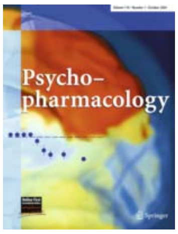

Conflict of Interest Disclosure Form

It is the policy of the *Psychopharmacology* to ensure balance, independence, objectivity, and scientific rigor in the Journal. All authors are expected to disclose to the readers any real or apparent conflict(s) of interest that may have a direct bearing on the subject matter of the article. This pertains to relationships with pharmaceutical companies, biomedical device manufacturers or other corporations whose products or services may be related to the subject matter of the article or who have sponsored the study.

The intent of the policy is not to prevent authors with a potential conflict of interest from publication. It is merely intended that any potential conflict should be identified openly so that the readers may form their own judgements about the article with the full disclosure of the facts. It is for the readers to determine whether the authors' outside interest may reflect a possible bias in either the exposition or the conclusions presented.

The corresponding author will complete and submit this form, along with his or her submission, to the responsible Editor on behalf of all authors listed below.

Article Title No antidepressant-like acute effects of bright light on emotional information processing in healthy volunteers

Authors Alexander Kaltenboeck, Tereza Ruzickova, Veronika Breunhölder, Tarek Zghoul, Philip Cowen, Catherine J Harmer

Please note that a conflict of interest statement is published with each paper.

I certify that there is no actual or potential conflict of interest in relation to this article. If any conflict exists, please define hereafter:

Conflict (if none, "None" or describe financial interest/arrangement with one or more organizations that could be perceived as a real or apparent conflict of interest in the context of the subject of this article):

Catherine J Harmer has received consultancy payments from P1vital Ltd., Zogenix, Sage Pharmaceuticals, J&J, Lundbeck and Pfizer.

All other authors report having no relevant conflicts of interest in relation to this work.

Name Alexander Kaltenboeck

Signature Alexander Kaltenboeck Date 29/04/2021

Please scan the completed and signed form and then submit it as an electronic file along with your manuscript. No manuscripts will be accepted for review without this form.
